# Editorial: Immunotherapy in Hepatocellular Carcinoma

**DOI:** 10.3389/fonc.2021.698515

**Published:** 2021-06-09

**Authors:** Bin Li, Ka-On Lam, Victor Ho-Fun Lee, Weijia Fang

**Affiliations:** ^1^ Department of Medical Oncology, First Affiliated Hospital, School of Medicine, Zhejiang University, Hangzhou, China; ^2^ Department of Clinical Oncology, Li Ka Shing Faculty of Medicine, The University of Hong Kong, Hong Kong, China

**Keywords:** hepatocellular carcinoma, immunotherapy, PD-1, liquid biopsy, tumor immune microenvironment, gut microbiota

Hepatocellular carcinoma (HCC) is the most common type of primary liver cancer with high incidence and mortality worldwide (1). Curative treatment options for patients with early-stage HCC remain reasonable, including surgical resection, ablation or transplantation (2). However, a large proportion of HCC patients are often diagnosed at advanced stage and with limited effective treatments (2). And the objective response rates and prognosis of advanced HCC treated with several recommended targeted therapies are still dismal only until recently (3–5). Newly-developed immunotherapies especially immune checkpoint inhibitors (ICIs) have definitely evolved the treatment strategy of advanced HCC, due to the encouraging efficacy and acceptable toxicity. Although this is a significant progress, further improvement is still an unmet need.

In this Research Topic, two comprehensive reviews introduce the clinical application of ICIs in the treatment of HCC and underlying clinical challenges, which draw a picture named “the past, present and future of HCC immunotherapy” (Donisi et al.; Zhang et al.).

Hepatitis B virus (HBV) infection is a primary risk factor for the development of HCC (6). And the clinical performance of ICIs in such particular population is still not very clear. A real-world study is carried out in an endemic area of HBV infection, which demonstrates acceptable toxicity and favorable efficacy of nivolumab monotherapy in unresectable HCC (Sung et al.). Of interest, there exist significant intratumoral heterogeneity and disturbed immune microenvironment in HCC. And the striking heterogeneous responses of multiple lesions from a single patient to nivolumab immunotherapy are also thoroughly studied. A retrospective analysis was carried out to evaluate the clinical outcomes of recurrent hepatitis B virus-related HCC who received nivolumab plus chemotherapy or targeted treatment (Chen et al.). After multiple lines of therapy, nivolumab-based therapy still displayed antitumor activity and there were less frequent treatment-related adverse events of any grade in recurrent HCC patients, even for with HBV infection.

Indeed, HCC is a notorious tumor. Although current immunotherapy has brought about better clinical outcomes, the improvements are often modest and the corresponding scope of clinical application is not fully optimized. Nowadays, various immunotherapy-based combination strategies have shown early promising anti-tumor activity (Donisi et al.). And more researches of combined immunotherapy are designed to deal with the complexity of HCC.

Locoregional therapies not only achieve local control in HCC but also could initiate an immune response by exposing neo-tumor-associated antigens *via* necrosis of the tumor cells (7). During programmed cell death protein 1 (PD-1) blockade immunotherapy, HCC patients achieve disease control or atypical progressive diseases (different responses in multi-lesions of the same individual). Thus, a proof-of-concept clinical trial was carried out to explore whether subtotal thermal ablation could increase the response rate of anti-PD-1 monotherapy and improve survival in this special population (Lyu et al.).

HCC is inclined to invade adjacent vasculature in particular the portal vein causing portal vein tumor thrombosis (PVTT) (8). Radiotherapy (RT), a standard option for HCC with PVTT, gradually change its role from a palliative treatment to a curative one. Thus, a randomized controlled study is designed by Hu et al. to confirm the efficacy and safety of combining stereotactic body RT (SBRT) with camrelizumab and apatinib in first line treatment for HCC patients with PVTT.

Not limited to selective combination, some new directions of immunotherapy are being discovered. Zhang et al. review gut microbiota and related potential treatment options for liver cancer. Certain bacterial species could improve anti-tumor immunity and enhance the efficacy of immunotherapy by modulating the components of bile acids. And modulating gut microbial components is considered to be a potential strategy to improve the efficacy of immunotherapy for HCC treatment.

Keeping the balance of cellular senescence is closely related to the occurrence and progression of HCC. With more in-depth research on cellular senescence, dual effects of cellular senescence and underlying mechanism of induced immune surveillance are gradually unmasked. A novel review by Liu et al. summarize the latest advances about hepatocellular senescence, and bring up some emerging intervention strategies in senescence-related therapy which HCCs that may benefit from tumor immune microenvironment remodeling. For instance, activating immune surveillance, recruiting functional immune cell types and eliminating atypical proliferative hepatocyte may act as the key elements of these senescence based “new immunotherapy”.

HCC is a highly aggressive disease with a poor prognosis and anti-PD-1 blockades prolong the median overall survival of advanced HCC to about 13-15 months (9, 10). On the basic of tumor immune microenvironment (TIME) phenotypes and differentially expressed gene clusters, Chen et al. construct a TIME score model. And further analysis reveals TIME score is positively associated with clinicopathologic features and somatic gene mutations. In another study of Chen et al., a nomogram constructed by potential prognostic factors including age, ECOG status, hepatectomy status, and transcatheter arterial chemoembolization (TACE) use, is performed to distinguish high-risk group and low-risk group of HCC patients. These prediction model certainly exhibit robust prognostic value for HCC. Moreover, Zhang et al. also reveal that specific group of bacteria or change of gut microbiome could be promising biomarkers used for diagnosis and prognosis of HCC.

Recently, more and more molecules and proteins have been unveiled, which are closely associated with the carcinogenesis and development of HCC as well as the tumor immune microenvironment. In this topic, Tang et al. summarize the major biological functions of circRNAs in liver cancer and emphasize the circRNAs-induced immune escape, by predominantly affecting natural killer cells.

These multi-angle articles collected in this Research Topic of Frontiers in Oncology, present an attractive scope of what is novel, promising, and controversial in the HCC immunotherapy field. We hope these valuable work would aid clinicians to understand and select immunotherapy options more wisely for the better management of HCC patients.

## Author Contributions

All authors contributed to the article and approved the submitted version.

## Conflict of Interest

The authors declare that the research was conducted in the absence of any commercial or financial relationships that could be construed as a potential conflict of interest.
